# *Phytophthora infestans* Dihydroorotate Dehydrogenase Is a Potential Target for Chemical Control – A Comparison With the Enzyme From *Solanum tuberosum*

**DOI:** 10.3389/fmicb.2019.01479

**Published:** 2019-06-28

**Authors:** Manuel F. Garavito, Heidy Y. Narvaez-Ortiz, Dania Camila Pulido, Monika Löffler, Howard S. Judelson, Silvia Restrepo, Barbara H. Zimmermann

**Affiliations:** ^1^Departamento de Ciencias Biológicas, Universidad de los Andes, Bogotá, Colombia; ^2^Laboratorio de Micología y Fitopatología, Universidad de los Andes, Bogotá, Colombia; ^3^Faculty of Medicine, Department of Biology, University of Marburg, Marburg, Germany; ^4^Department of Microbiology and Plant Pathology, University of California, Riverside, Riverside, CA, United States

**Keywords:** dihydroorotase dehydrogenase, inhibitors, pyrimidine biosynthesis, *Phytophthora infestans*, *Solanum tuberosum*, kinetics, A77 1726

## Abstract

The oomycete *Phytophthora infestans* is the causal agent of tomato and potato late blight, a disease that causes tremendous economic losses in the production of solanaceous crops. The similarities between oomycetes and the apicomplexa led us to hypothesize that dihydroorotate dehydrogenase (DHODH), the enzyme catalyzing the fourth step in pyrimidine biosynthetic pathway, and a validated drug target in treatment of malaria, could be a potential target for controlling *P. infestans* growth. In eukaryotes, class 2 DHODHs are mitochondrially associated ubiquinone-linked enzymes that catalyze the fourth, and only redox step of *de novo* pyrimidine biosynthesis. We characterized the enzymes from both the pathogen and a host, *Solanum tuberosum*. Plant DHODHs are known to be class 2 enzymes. Sequence analysis suggested that the pathogen enzyme (PiDHODHs) also belongs to this class. We confirmed the mitochondrial localization of GFP-PiDHODH showing colocalization with mCherry-labeled ATPase in a transgenic pathogen. N-terminally truncated versions of the two DHODHs were overproduced in *E. coli*, purified, and kinetically characterized. StDHODH exhibited a apparent specific activity of 41 ± 1 μmol min^-1^ mg^-1^, a k_cat_^app^ of 30 ± 1 s^-1^, and a K_m_^app^ of 20 ± 1 μM for L-dihydroorotate, and a K_m_^app^= 30 ± 3 μM for decylubiquinone (Qd). PiDHODH exhibited an apparent specific activity of 104 ± 1 μmol min^-1^ mg^-1^, a k_cat_^app^ of 75 ± 1 s^-1^, and a K_m_^app^ of 57 ± 3 μM for L-dihydroorotate, and a K_m_^app^ of 15 ± 1 μM for Qd. The two enzymes exhibited different activities with different quinones and napthoquinone derivatives, and different sensitivities to compounds known to cause inhibition of DHODHs from other organisms. The IC_50_ for A77 1726, a nanomolar inhibitor of human DHODH, was 2.9 ± 0.6 mM for StDHODH, and 79 ± 1 μM for PiDHODH. *In vivo*, 0.5 mM A77 1726 decreased mycelial growth by approximately 50%, after 92 h. Collectively, our findings suggest that the *Pi*DHODH could be a target for selective inhibitors and we provide a biochemical background for the development of compounds that could be helpful for the control of the pathogen, opening the way to protein crystallization.

## Introduction

Within the Phylum Oomycota, the species of the genus *Phytophthora* are all considered devastating pathogens of crops and landscape plants, and are responsible annually for huge economic losses worldwide (Lamour et al., 2007; Attard et al., 2008). *Phytophthora infestans* causes late blight disease in potato, tomato, and other solanaceous crops. In these hosts, the entire plant is destroyed within a few days after the first lesions are observed (Fry, 2008).

Despite their economic importance, *Phytophthora* species remain poorly characterized at the biochemical level. The enzyme dihydroorotate dehydrogenase (DHODH, E.C. 1.3.5.2), catalyzing the fourth step in *de novo* pyrimidine biosynthesis has been attractive for drug development for decades, with over 100 compounds that inhibit its activity in diverse organisms (Munier-Lehmann et al., 2013). In most eukaryotes, including plants (Witz et al., 2012) and some plant pathogenic fungi (Zameitat et al., 2007), class 2 DHODHs are associated with the outer surface of the inner mitochondrial membrane (Rawls et al., 2000) and transfer electrons from dihydroorotate oxidation to ubiquinone in the respiratory chain. Class 2 DHODHs are also found in gram-negative bacteria (Björnberg et al., 1999), anchored to the inner side of the periplasmic side of the inner cytoplasmic membrane, similarly transferring electrons to the respiratory chain.

Human DHODH, has been intensively studied, and is the target of A77 1726, also known as teriflunomide, the active metabolite of leflunomide, used in the treatment of rheumatoid arthritis (Fragoso and Brooks, 2015), and multiple sclerosis (Faissner and Gold, 2018). Recent work suggests that inhibitors of this enzyme show promise in the treatment of different cancers (Sykes et al., 2016). Structural differences between the human enzyme and pathogen enzymes have been exploited to develop species-specific inhibitors. For example, DSM265, a triazolopyrimidine-based compound targeting DHODH from the apicomplexan parasite *Plasmodium falciparum*, is currently undergoing phase 2 clinical trials (Ashley, 2017; McCarthy et al., 2017). Similarly, the antifungal properties of F901318, an inhibitor of DHODHs from pathogenic *Aspergillus* sp., is being tested in phase 1 trials (Oliver et al., 2016). Oomycete DHODH has been evaluated as an enzymatic target in biochemical screenings, leading to the identification of compounds that display *in vitro* inhibition of *Pythium aphanidermatum* DHODH, and *in vivo* whole plant control of *Plasmopara viticola* (Parker et al., 2002). Interestingly, gene deletion simulations in *P. infestans* by Rodenburg et al. (2018) identified the DHODH as one of 72 genes essential for growth, thus warranting further study.

Collectively, these observations suggest that selective inhibitors could be developed for *P. infestans* DHODH that would exert little or no effect on the host, or the human consumer. In the present study we produced recombinant pathogen (PiDHODH) and *Solanum tuberosum* (StDHODH) enzymes, measured their catalytic properties, and demonstrated differences in their activities with electron acceptors and in their sensitivities to inhibitors. We showed that PiDHODH has a micromolar IC_50_ for A77 1726, that is ≈37-fold lower than that of the host enzyme, and demonstrated that this compound inhibits growth of *Phytophthora in vivo*. Our work highlights the potential of PiDHODH as a possible target for developing novel combination strategies for pathogen control.

## Materials and Methods

### Reagents

Reagents were purchased from Sigma–Aldrich unless otherwise specified, as follows. teriflunomide (A77 1726) Sanofi Aventis, Germany; ametoctradin (Initium) BASF, Germany; atovaquone (GlaxoSmithKline, United Kingdom); dichloroallyl lawsone (DCL/NSC-73735) NIH, United States; 2-hidroxy-1,4-naphthoquinone (lawsone) Aldrich, Germany; toltrazuril, Bayer AG, Germany. Several compounds were obtained from the Chemotherapuetic Agents Repository of the National Cancer Institute (compounds preceded by NSC). *N*-(3,5-difluoro-4-(trifluoromethyl)phenyl)-5-methyl-[1,2,4]triazolo[1,5-a]pyrimidin-7-amine (DSM-190) was the gift of Dr. M. A. Phillips, The University of Texas at Austin, United States (Baldwin et al., 2005). *N*-(3-chlorobiphenyl-4-yl)-2-cyano-3-hydroxybut-2-enamide (MD-241, compound 28) (Davies et al., 2009); *N*-(3-chloro-2-methoxybiphenyl-4-yl)-2-cyano-3-hydroxybut-2-enamide (MD-209, compound 19) (Davies et al., 2009); 2-(4-biphenylylcarbamoyl)-1-cyclopentene-1-carboxylic acid (MD-108, compound 1) (Leban et al., 2005); 2-(4-biphenylylcarbamoyl)-2-benzoic acid (MD-129, compound 2) (Heikkilä et al., 2006); were the gift of Drs. Johnson and Fishwick, University of Leeds, United Kingdom. Restriction enzymes and other cloning enzymes were from New England Biolabs, United States. Curzate^®^ M8 was obtained from Dupont.

### Sequence Analysis

**FIGURE 1:**
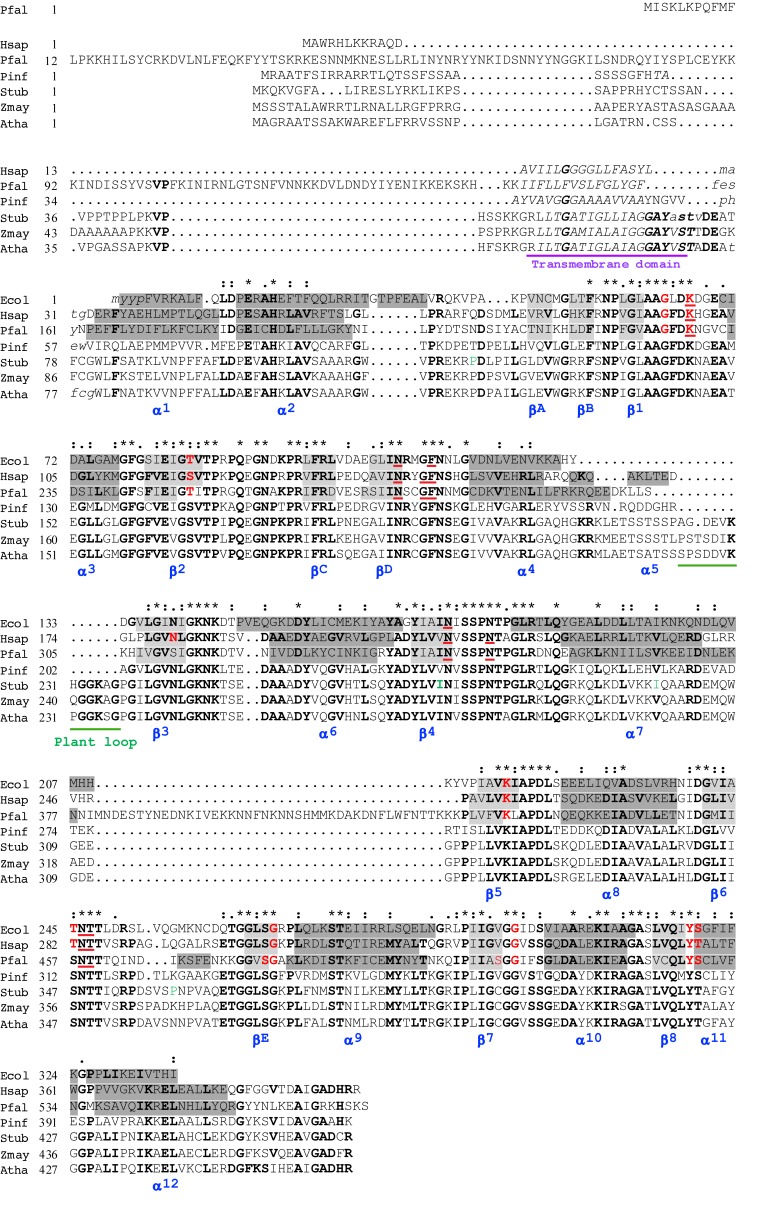
Alignment of the predicted amino acid sequences of *Phytophthora infestans* and *Solanum tuberosum* DHODHs with other class 2 DHODHs. Selected class 2 DHODHs were aligned according to sequences and 3D-structures of human, 1D3H (Liu et al., 2000), *P. falciparum*, 1TV5 (Hurt et al., 2006), and *E. coli*, 1F76 (Nørager et al., 2002) DHODHs. Secondary structures are named (blue) according to Liu and co-workers, where alpha helices are highlighted in dark gray, and beta sheets in light gray (Liu et al., 2000). The transmembrane domain is underlined in purple, and a sequence found only in plants is underlined in green. In the human DHODH sequence, residues interacting with FMN are shown in red, and residues interacting with orotate are underlined in red (Liu et al., 2000). Conserved residues are indicated with asterisks, conservative substitutions with colons, and semiconservative substitutions with periods, above the sequences. Residues in bold are conserved in more than 55% of the sequences. Four residues (P120T, I273V, I299V, and P359Q), shown in green, indicate positions where the published StDHODH sequence (PGSC0003DMG401016396) differs from the sequence of ΔN69StDHODH (P120T, I273V, I299V, and P359Q). Ecol, *E. coli*, 1F76 – WP_001295934; Hsap, *Homo sapiens*, 1D3G – NP_001352; Pfal, *P. falciparum*, 1TV5 – XP_966023; Pinf, *P. infestans*, PITG_01913; Stub, *S. tuberosum*, PGSC0003DMG401016396; Zmay, *Zea mays*, NP_001152058; Atha, *A. thaliana*, AAN64025.

Sequences coding for the enzymes were retrieved from the genomes of *P. infestans* (strain T30-4), BioProject accession number PRJNA17665, and *S. tuberosum*, EnsemblPlants^1^. Additional sequences were retrieved from National Center for Biotechnology Information (NCBI). Multiple sequence alignments were performed according to sequence and 3D-structure using the STRAP program (default parameters)^2^. In Figure 1, the amino acid sequences of the secondary structure elements in three proteins with crystallographic structures are identified as specified in the relevant publications as follows: *E. coli* DHODH (Nørager et al., 2002), *P. falciparum* DHODH (Deng et al., 2009), human DHODH (Liu et al., 2000). Different designations for the alpha helices and beta sheets are used in these three publications; in Figure 1 we use the designations of Liu and coworkers. Mitochondrial targeting and signal peptide sequences were predicted with Mitoprot II 1.101 (default parameters) (Claros and Vincens, 1996), and TargetP 1.1 (plant network) (Nielsen et al., 1997; Emanuelsson et al., 2000), and MitoFates (plant) (Fukasawa et al., 2015). The N-terminal transmembrane domains were predicted by HMMTOP (default parameters) (Tusnady and Simon, 2001).

### Expression Constructs

*Solanum tuberosum* cDNA was prepared from commercially available potato plants (Sabanera variety). Total RNA was extracted from frozen mycelia or from 300 mg of plant leaves and cDNA was synthesized (Garcia-Bayona et al., 2014). Subsequently, a fraction of the cDNA reactions (2 μL) was used for PCR amplification. cDNA prepared from *P. infestans* strain 1043, a Colombian isolate from potato with A1 mating type, and also belonging to the EC-1 clonal lineage, was used for the cloning (Vargas et al., 2009). The isolates were cultured routinely on rye agar medium and incubated at 19°C for 9 days in the dark (Goodwin et al., 1998). Mycelia were collected from two plates and frozen until use.

Primers for PCR amplification were designed based on the sequences Gene ID PGSC0003DMG401016396 (StDHODH) and PITG_01913 (PiDHODH) (Supplementary Table 1). PCR products were adenylated and ligated in the pGMT-Easy vector (Promega, United States), prior to transforming in *E. coli* DH5-α electro-competent cells (Agilent, United States). The cloned inserts were confirmed by sequencing (Macrogen, South Korea). Recombinant plasmids containing the coding sequences, were digested with *Nde*I-*Xho*I (StDHODH) or *Bam*HI (PiDHODH), separated by 1% agarose gel electrophoresis, purified (BIO-101, United States) and ligated into the pET-15b (StDHODH) or pET-19b (PiDHODH) expression vectors (Novagen, United States) that had been similarly treated. Linearized pET19b was treated with calf intestine alkaline phosphatase (Invitrogen, United States) prior to ligation. Constructs were transformed into *E. coli* BL21CodonPlus(DE3) electro-competent cells with a Micropulser Electroporator (BioRad, United States). The N-terminally truncated version of StDHODH (ΔN69StDHODH) started at residue 70 (ASTV), and the N-terminally truncated version of PiDHODH (ΔN54PiDHODH) sequence started at residue 55 (PHEW). The recombinant proteins expressed from the pET19b vector had a 26 residue N-terminal tag including 10-His, and recombinant proteins expressed from pET15b had a 19 residue N-terminal tag including 6-His. The published StDHODH sequence (PGSC0003DMG401016396) differed from the ΔN69StDHODH sequence in four positions (P120T, I273V, I299V, and P359Q).

### Expression and Purification

Transformed *E. coli* BL21-CodonPlus(DE3)-RP electrocompetent cells were precultured overnight in Luria-Bretani broth medium (LB) with 100 μg/mL ampicillin as the selection marker at 37°C with 200 rpm agitation. The preculture was diluted to 5% in LB containing 100 μg/mL ampicillin and grown at 37°C until OD_600nm_ = 0.5–0.6. Induction was performed with 1 mM isopropyl β-D-thiogalactopyranoside (IPTG), supplemented with 0.1 mM flavin mononucleotide (FMN) and growth was continued overnight at room temperature (Baldwin et al., 2002). Induced cultures were harvested by centrifugation at 3,500 × *g* for 15 min at 4°C, supernatant discarded and cell pastes were frozen at -80°C until use.

For the purification of the recombinant proteins, a pellet from 500 mL of bacterial cell culture was resuspended in Buffer A [50 mM Tris-HCl pH 8, 300 mM NaCl and 10% glycerol, 5 mM imidazole, 1 mM phenylmethanesulfonyl fluoride (PMSF) and 1 mM benzamidine] containing 2% Triton X-100. The cell suspension was incubated with lysozyme (1 mg/mL) on ice for 2 h. Cells were disrupted by sonication on ice (30 cycles of 20 s each, output control setting of 8 and 100% duty cycle) using a 250 Analog Sonifier (Branson). After sonication, insoluble and soluble fractions were separated by centrifugation (8,500 × *g*, 1 h, 4°C). The recombinant proteins were purified from the clarified cell lysates using Co^2+^ affinity columns (Thermo Scientific) equilibrated with Buffer A containing 0.5% Triton X-100 following the manufacturer’s recommendations, and eluted with buffer A containing 250 mM imidazole. The yields of purified protein were 2.0 mg/L of cell culture for StDHODH, and 1.3 mg/L of cell culture for PiDHODH. The expected sizes for the truncated recombinant proteins were: PiDHODH 43.1 kDa and StDHODH 43.7 kDa.

For enzymatic activity assays, fractions were subjected to buffer exchange performed with PD-10 columns (Sephadex G-25-M, GE Healthcare) with buffer (50 mM Tris-HCl pH 8, 150 mM NaCl, 0.1% Triton X-100 and 10% glycerol) (Ullrich et al., 2001).

### SDS-PAGE and Electrotransfer

Protein samples were fractionated by SDS-PAGE on 12% running gels, with 5% stacking gels. Electrophoresis was performed in a BioRad Mini-Protean II electrophoresis cell for 1 h, at 200 volts, constant voltage. Gels were visualized by staining with Coomassie Blue G-250 dye.

### Enzymatic Assays and Kinetic Analysis

In our standard activity assay we monitored the reduction of 2,6-dichlorophenol-indophenol (DCIP) at 600 nm (ε = 18,800 M^-1^ cm^-1^) at 30°C in a reaction buffer containing 50 mM Tris-HCl pH 8.0, 150 mM KCl, 0.1% Triton X-100, 10% glycerol, 1 mM L-dihydroorotate, 0.1 mM DCIP, 0.1 mM decylubiquinone (Qd) (Zameitat et al., 2007), with concentrations of 19 nM (StDHODH) or 6.6 nM (PiDHODH). The apparent kinetic constants of the substrates were determined by varying L-dihydroorotate concentration (2.5–1,250 μM) while keeping Qd constant at 100 μM, or by varying Qd (1.25–200 μM) at a fixed dihydroorotate concentration of 1 mM. The kinetic data were evaluated by fitting the data to the Michaelis–Menten equation *v = Vmax*^∗^[S]/ (Km + [S]) using GraphPad Prism v7 software. The apparent *kcat* was calculated from *kcat = Vmax*/[ET], where [ET] is total enzyme concentration, based on one active site monomer. Background oxidase activities due to direct reduction of DCIP by the enzyme in the absence of Q_d_ were subtracted from the activities measured in the presence of Q_d_. For StDHODH the background oxidase activity was 9 ± 2 μmol min^-1^ mg^-1^, and for PiDHODH it was 6 ± 1 μmol min^-1^ mg^-1^.

To test alternative electron acceptors, stock solutions of 10–50 mM quinones, napthoquinones, and benzoquinones were prepared in absolute ethanol. Enzyme activities were measured with the standard assay described above, using 0.1 mM of the acceptor in place of Qd.

Stock solutions of all inhibitors were prepared in reaction buffer, or in dimethyl sulfoxide (DMSO). Activities were measured with the standard DCIP assay with saturating concentrations of Qd (0.1 mM) and dihydroorotate (1 mM), in the presence of 0.5 mM inhibitor. The final concentrations of 5% DMSO used in the assay solutions were found not to affect the DHODH activity of either recombinant enzyme. DHODH activities were measured by the standard assay.

Protein concentration was measured using the bicinchoninic assay (Pierce) with bovine serum albumin as the standard.

Absorbance was measured in microplates with a Thermo Scientific Multiskan GO UV/visible spectrophotometer.

### *P. infestans* Localization Constructs, Transformation, Selection, and Imaging

To determine the subcellular localization of PiDHODH, we made a construct fused on the 3′-ends to eGFP. The desired sequences were amplified by PCR using primer pairs (Supplementary Table 1) which introduced a *Pac*I site at the 5′ end and an *Nhe*I site at the 3′ end. The fragment was ligated to the pGEM-T Easy vector (Promega) and transformed in *E. coli* DH5-α chemiocompetent cells. The pGEM-T Easy plasmid was subjected to double restriction *Pac*I-*Nhe*I, the fragment was subcloned in pGFPH (Ah-Fong and Judelson, 2011), and confirmed by sequencing. The resulting fusion construct was expressed under the control of the promoter and terminator from the *Ham34* gene of *Bremia lactucae*. A control plasmid, pATPase-mCherryN, was used to visualize mitochondria (Ah-Fong and Judelson, 2011). Plasmid DNA for transformation was obtained using the Plasmid DNA Purification Kit NucleoBond PC 500 (Macherey-Nagel, Germany). Stable transformants were generated with isolate 1306 (A1 mating type, United States isolated from tomato) (Judelson et al., 1995), using the modifications proposed by Ah-Fong and Judelson (2011) to the original protoplast method (Judelson et al., 1991). For co-transformation, a total of 40 μg of plasmid DNA were used per experiment; 20 μg of the localization plasmid (PiDHODH-eGFP) and 20 μg of the control plasmid which expresses the b-subunit of the mitochondrial ATPase N-terminally fused to mCherry (pATPase-mCherryN). Transformed strains were grown with 30 μg mL^-1^ hygromycin and 10 μg mL^-1^ G418.

GFP- and mCherry-expressing strains were preselected by a visual inspection of the transformants by confocal laser scanning microscope using a Leica TCO-SP2. Transformants were grown in Petri dishes of rye-sucrose media for 6–8 days, mycelia from the external edge of the growth radius were mounted on drops of 10 μL of water in glass slides and sealed with transparent nail polish. GFP expression was visualized using 40X/0.8 or 63X/0.9 objectives with excitation/emission settings 488 nm/500–550 nm for GFP 543 nm/575–700 nm for mCherry. Fluorescent sporangia were obtained after 7–10 days.

### Bioassays

The isolate Z3-2 of *P. infestans*, with EC-1 clonal lineage, and A1 mating type, was grown for 8 to 10 days at 18°C on rye-sucrose agar plates plus 50 μg/mL ampicillin. Plates containing sporulated mycelia were flooded with ultra pure water and rubbed with a glass rod to liberate the sporangia. The sporangia were purified from the suspension by filtration through a 50 μm nylon mesh, and counted with a hemocytometer. Then 4 × 10^6^ sporangia/mL were incubated for 2–4 h at 10°C to induce the liberation of free-swimming zoospores. The suspension containing zoospores and sporangia (125 μL) was dispensed into five replicate wells of a 96-well flat bottom tissue culture treated plates, and combined with 75 μL of Henniger media (Henniger, 2007) and 25 μL of each compound tested. A77 1726 was resuspended in DHODH reaction buffer, and curzate was resuspended in water. Microtiter plates were covered and placed in the dark at 18°C. Growth was evaluated by measuring the OD_610nm_ at 20–24 h intervals for 100 h with a Multiskan^TM^ GO Microplate Spectrophotometer (Thermo Scientific). Data analysis and graphs were performed using GraphPad Prism Software v7 for Mac ^3^.

## Results

### StDHODH and PiDHODH Predicted Amino Acid Sequences Are Most Similar to Class 2 Enzymes

The gene encoding the StDHODH was identified as a single copy protein (Gene ID PGSC0003DMG401016396) in the *S. tuberosum* genome assembly, EnsemblPlants^1^. It had an open reading frame (1275 bp) comprised of 11 exons, which encoded a 48.9 kDa protein (460 residues) with an isoelectric point of 9.35 consistent with the high pI predicted for other class 2 DHODHs. The derived amino acid sequence contained typical features of the class 2 DHODHs, including an N-terminal extension, a transmembrane domain, a conserved serine (S277) that aligned with the catalytic serines of *E. coli* (S175), human (S215), and *P. falciparum* (S345) DHODHs, and conserved residues for binding the electron acceptor, FMN, and orotate (Figure 1). The identity with other plant DHODHs was high (*Zea mays* 73%, *A. thaliana* 77%), and revealed that the enzyme was more similar to the class 2 DHODHs (*Homo sapiens* 49%*, P. falciparum* 30%, *P. infestans* 48%, *E. coli* 41%) (Supplementary Table 2) than to class 1 DHODHs (*T. cruzi* 21%, *S. cerevisiae* 21%). The N-terminal extension found in plant DHODHs was longer than the extensions found in the human or the oomycete enzymes, but shorter than that found in apicomplexan enzymes. A cleavage site at residue 28 was predicted by both TargetP 1.1 (Nielsen et al., 1997; Emanuelsson et al., 2000) and MitoFates (Fukasawa et al., 2015). The StDHODH displayed a small insertion of 13 residues between positions 225 and 236 that was also present in other plant DHODHs, but not in other eukaryotic class 2 enzymes. Four positions in our cloned sequence of StDHODH (P120T, I273V, I299V, and P359Q) differed from the published sequence shown in Figure 1. Probably these differences are not significant, since all of these residues are found in other sequences shown in the alignment, for example, threonine in position 120 is observed in PiDHODH, valine in position 273 is observed in HsDHODH, valine in position 299 appears in all the eukaryotic DHODHs in Figure 1, and glutamine in position 359 is found in HsDHODH. The last of these differences is in a poorly conserved region of the sequence. Furthermore, it is relevant that cultivated *S. tuberosum* is a highly heterogenous autotetraploid (Manrique-Carpintero et al., 2018), and thus possesses four alleles of DHODH, which may exhibit sequence differences.

The gene encoding PiDHODH was identified as a single copy, without introns, in supercontig 2 (locus: PITG_01913) in the *P. infestans* genome. The open reading frame of 1275 bp encoded a 45.3 kDa protein (424 residues) with an isoelectric point of 8.74. The predicted amino acid sequence contained the class 2 DHODH N-terminal extension, transmembrane domain, conserved serine (S242), and conserved FMN and orotate binding residues. The percent identity shared with other DHODHs showed that the *P. infestans* enzyme was more similar to the class 2 enzymes (*H. sapiens* 53%, *A. thaliana* 46%, *S. tuberosum* 48%, *E. coli* 38%, *P. falciparum* 31%), than to class 1 enzymes (*T. cruzi* 22%, *S. cerevisiae* 19%) (Supplementary Table 2). As expected, alignments revealed that the DHODH from *P. infestans* shared the highest identity percentages with the other four *Phytophthora* species (*P. parasitica* 93%, *P. capsici* 90%, *P. ramorum* 87%, *P. sojae* 87%) and lower identity percentages compared to other oomycetes (*Plasmopara halstedii* 84%, *Pythium ultimum* 70%, *Saprolegnia parasitica* 60%, *Aphanomyces invadans* 57%) (Supplementary Figure 1).

### PiDHODH Is Targeted to Mitochondria

**FIGURE 2:**
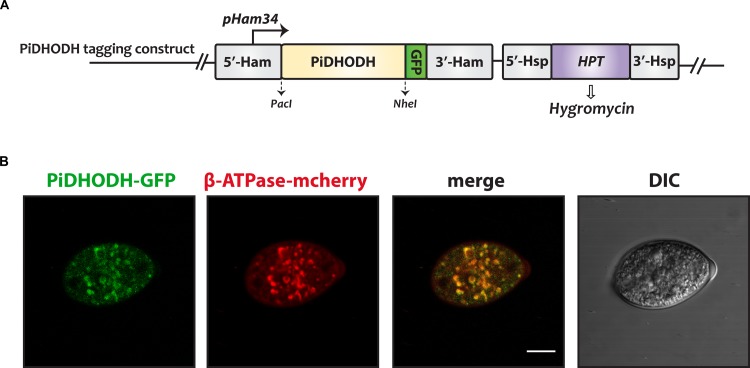
The subcellular localization of *P. infestans* DHODH in sporangia. **(A)** The construct used to produce DHODH fused on the C-terminus to green fluorescent protein (GFP). **(B)** A sporangium doubly transformed with GFP-labeled PiDHODH (green), and with the mitochondrial marker, ATP synthase fused to mCherry (red) (Ah-Fong and Judelson, 2011) is shown. DIC, differential interference contrast. Scale bar: 10 μm.

The PiDHODH contains a predicted cationic mitochondrial targeting sequence (residues 1–23) at the N-terminus, followed by a predicted hydrophobic transmembrane helix sequence (residues 30–49). A proteolytic cleavage site was predicted at position 23 by MitoprotII v1.101. To confirm the mitochondrial localization we constructed an eGFP PiDHODH fusion, and co-transformed *P. infestans* with an mCherry tagged ATP synthase construct. PiDHOD was found to colocalize with the ATP synthase in sporangia (Figure 2).

### ΔN69StDHODH and ΔN54PiDHODH Recombinant Proteins Are Produced in Soluble Form in *E. coli*

Class 2 DHODHs from several organisms have been produced as recombinant proteins, and in most cases these expression constructs eliminate the N-terminal sequence, which contains the hydrophobic transmembrane anchor. The elimination of these N-terminal extensions appear to exert relatively small effects on enzyme activity (Baldwin et al., 2002; Zameitat et al., 2006).

We expressed both full-length (data not shown) and N-terminally truncated recombinant proteins of StDHODH, with N-terminal polyhistidine-tags to facilitate purification. While the full-length StDHODH was found in the insoluble fraction, the truncated version, ΔN69StDHODH, was expressed in soluble form, and was purified (Supplementary Figure 2). Interestingly, both full-length and truncated versions of *A. thaliana* DHODH were soluble and active, although the polyhistidine-tags in these recombinant proteins were located at the C-termini (Ullrich et al., 2002). PiDHODH was expressed using a similar strategy, and only the truncated version ΔN54PiDHODH was soluble and could be purified (Supplementary Figure 2).

### Apparent Kinetic Parameters of ΔN69StDHODH and ΔN54PiDHODH

**FIGURE 3:**
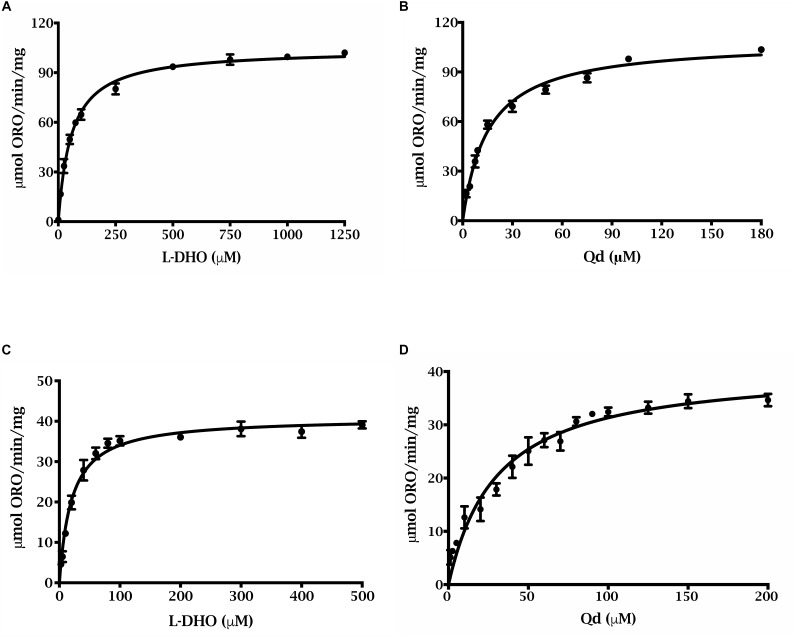
Steady state kinetics of of purified truncated recombinant *P. infestans* and *S. tuberosum* DHODHs. ΔN54PiDHODH saturation curves for **(A)** L-dihydroorotate and **(B)** decylubiquinone are shown. ΔN69StDHODH saturation curves for **(C)** L-dihydroorotate and **(D)** decylubiquinone are shown. All curves were fit to the Michaelis–Menten equation, v = (V_max_ ⋅ [S])/(K_m_ + [S]).

Preliminary kinetic data were collected for the N-terminally truncated DHODHs (Figure 3), and the apparent kinetic parameters were compared to those of other DHODHs (Table 1) (Ullrich et al., 2001; Hortua Triana et al., 2012). ΔN69StDHODH exhibited an apparent specific activity of 41 ± 1 μmol min^-1^ mg^-1^, a k_cat_^app^ of 30 ± 1 s^-1^, a K_m_^app^ of 20 ± 1 μM for L-dihydroorotate, and a K_m_^app^ of 30 ± 3 μM for decylubiquinone (Qd). ΔN54PiDHODH exhibited an apparent specific activity of 104 ± 1 μmol min^-1^ mg^-1^, a k_cat_^app^ of 75 ± 1 s^-1^, a K_m_^app^ of 57 ± 3 μM for L-dihydroorotate, and a K_m_^app^ of 15 ± 1 μM for Qd.

**Table 1:** Apparent kinetic parameters of DHODHs from different organisms.

Organism	V_max_^app^ (μmol min^-1^ mg^-1^)	K_m_^app^ (μM DHO)	K_m_^app^ (μM Q_d_)	k_cat_^app^ (s^-1^)	k_cat_^app^/K_m_^app^ (DHO, x 10^6^ M^-1^ s^-1^)	k_cat_^app^/K_m_^app^ (Q_d_, x 10^5^ M^-1^ s^-1^)	References
*H. sapiens*	–	10	14	75	7.5	54	Ullrich et al., 2001
*R. norvegicus*	–	11	7	95	8.6	136	
*T. gondii*	–	60	29	89	1.5	31	Hortua Triana et al., 2012
*S. tuberosum*	41 ± 1	20 ± 1	30 ± 3	30 ± 1	1.5 ± 0.1	1.0 ± 0.1	This report
*P. infestans*	104 ± 1	57 ± 3	15 ± 1	75 ± 1	1.3 ± 0.1	49 ± 4	This report


*Enzyme activities were measured using the DCIP assay, at 30°C, pH 8.0, in 50 mM Tris-HCl, 150 mM KCl, and 0.1% Triton X-100. Glycerol at 10% was included in the assays of the *T. gondii, S. tuberosum*, and *P. infestans* DHODHs. Dihydroorotate was fixed at 1 mM with variable Q_d_ concentrations, and Q_d_ was fixed at 100 μM with variable dihydroorotate concentrations.*

We evaluated the enzyme activities with a variety of natural and artificial electron acceptors. Activities are expressed as a percentage, taking the activity measured with Qd as 100%. ΔN69StDHODH, exhibited the highest activities for coenzyme Qs having isoprenyl side chain lengths ranging from Q1 to Q9. ΔN54PiDHODH showed a preference for a slightly shorter range of isoprenyl side chain lengths, from Q1 to Q6. The two enzymes exhibited different activities for six derivatives of napthoquinone (Table 2).

**Table 2:** Activities of StDHODH and PiDHODH with alternative electron acceptors.

Co-substrate	Activity % StDHODH	Activity % PiDHODH
Decylubiquinone (Qd)	100 ± 7	100 ± 2
Ubiquinone (Q0)	96 ± 9	56 ± 1
Ubiquinone (Q1)	124 ± 11	111 ± 1
Ubiquinone (Q2)	123 ± 4	Not done
Ubiquinone (Q6)	102 ± 3	144 ± 2
Ubiquinone (Q9)	111 ± 2	66 ± 5
Ubiquinone (Q10)	19 ± 4	30 ± 1
2,5-Dimethyl-*p*-benzoquinone	67 ± 6	48 ± 1
5,8-Dihydroxy-1,4-naphthoquinone	113 ± 7	17 ± 3
1,4-Naphthoquinone	78 ± 5	50 ± 1
2-Hydroxy-1,4-naphthoquinone (lawsone)	14 ± 7	5 ± 1
5-Hydroxy-1,4-naphthoquinone (juglone)	5 ± 1	15 ± 1
5-Hydroxy-2-methyl-1,4-naphthoquinone (plumbagin)	46 ± 6	6 ± 1
2-Methyl-1,4-naphthoquinone (menadione, vitamin K3)	17 ± 7	23 ± 1
Ferricyanide	3.5 ± 0.4	1.6 ± 1.0


*Activities were measured with the standard DCIP assay using 0.1 mM of the electron acceptor indicated, and 1 mM dihydroorotate. The activity with Qd was set as 100%.*

### StDHODH and PiDHODH Show Differential Sensitivities to Inhibitors

**FIGURE 4:**
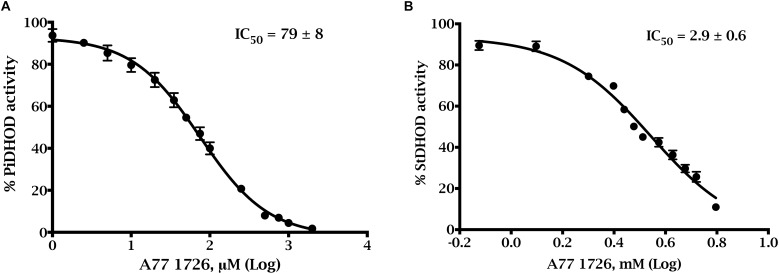
Dose-response inhibition curves of A77 1726 for truncated recombinant *P. infestans* and *S. tuberosum* DHODHs. Activities were measured with the standard DCIP assay with saturating concentrations of Qd (0.1 mM) and dihydroorotate (1 mM), in the presence of **(A)** 0–2,000 μM A77 1726 for ΔN54PiDHODH, and **(B)** 0–5.26 mM A77 1726 for ΔN69StDHODH.

We studied the susceptibilities of the *S. tuberosum* and *P. infestans* recombinant enzymes to various compounds, which have been demonstrated to be inhibitors of DHODHs in other species (Knecht et al., 2000; Baldwin et al., 2002; Leban et al., 2005; Heikkilä et al., 2006; Davies et al., 2009; Munier-Lehmann et al., 2013). We also tested atovaquone (Munier-Lehmann et al., 2013) and amectoctradin (Dreinert et al., 2018), two inhibitors that bind to ubiquinone sites in cytochrome bc_1_. The first two compounds in Table 3 have similarity to the substrate (dihydroorotate), or to the product (orotate), and the remaining compounds are expected to bind in the electron acceptor (ubiquinone) site. Nine of the compounds decreased PiDHODH activity, and six decreased StDHODH activity to below 50% of the activity observed in absence of inhibitor (Table 3). Differential inhibition for StDHODH and PiDHODH was observed for compounds A77 1726, toltrazuril, MD108, MD241, and NSC61890. The greatest difference in inhibition was observed for A77 1726, where PiDHODH activity was decreased to 14%, while StDHODH activity was unaffected. This difference was confirmed by the IC_50_ values (Figure 4) measured for A77 1726, 2.9 ± 0.6 mM for StDHODH, and 79 ± 8 μM for PiDHODH.

**Table 3:** Activities of StDHODH and PiDHODH in the presence of potential inhibitors.

Inhibitor	Activity % StDHODH	Activity % PiDHODH
5′-Fluorotic acid (5′-FOA)	55 ± 4	83 ± 3
Alloxan	74 ± 20	112 ± 4
A77-1726	97 ± 6	14 ± 3
Dichloroallyl lawsone (DCL)	39 ± 6	30 ± 2
Toltrazuril (1 mM)	0	67 ± 4
Brequinar	49 ± 1	98 ± 3
Redoxal	39 ± 3	31 ± 8
DSM190	38 ± 5	36 ± 10
MD 108	90 ± 4	23 ± 4
MD 129	95 ± 8	103 ± 2
MD 209	0	2.0 ± 0.5
MD 241	87 ± 3	1.0 ± 0.7
NSC 61890	36 ± 2	93 ± 2
NSC 71097	70 ± 10	129 ± 4
NSC 277965	130 ± 8	60 ± 2
Atovaquone	75 ± 6	127 ± 39
Ametoctradin	102 ± 5	41 ± 1


*Activities were measured with the standard DCIP assay with saturating concentrations of Qd (0.1 mM) and dihydroorotate (1 mM), in the presence of 0.5 mM inhibitor, unless otherwise indicated. The activity without inhibitor was set as 100%. Compounds that increase activity above 100% apparently acted as electron acceptors instead of inhibitors.*

### A77 1726 Inhibits the Growth of *P. infestans*

**FIGURE 5:**
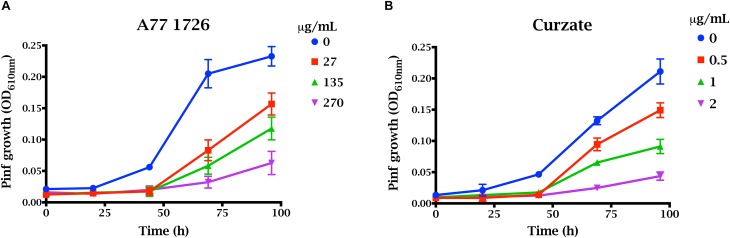
Effect of A77 1726 on the growth of *P. infestans*. Growth of zoospores and sporangia were evaluated by OD_610__nm_ for 100 h in the presence of **(A)** 0, 27, 136, 277 μg/mL (0, 0.1, 0.5, and 1 mM) A77 1726, or **(B)** 0, 0.5, 1, and 2 μg/mL Curzate.

We measured the growth of *P. infestans* sporangia and zoospores in liquid culture in the presence of PiDHODH inhibitor A77 1726 (Figure 5). For comparison, growth was measured in the presence of Curzate^®^ M8, an oomyceticide containing 64% w/w mancozeb [manganese zinc ethylenebis(dithiocarbamate)] and 8% w/w cymoxanil, with the former targeted to enzymes dependent on active sulfhydryl groups (Gullino et al., 2010), and the latter implicated in amino acid synthesis (Tellier et al., 2008). Of the two treatments, Curzate^®^ M8, is more effective, requiring only 1 to 2 μg/mL compared to 136 μg/mL (0.5 mM) required for A77 1726 to reduce the growth of the oomycete by approximately 50% at 92 h. Thus, although PiDHODH is approximately 40-fold more sensitive to A77 1726 compared to StDHODH, the compound is still a relatively poor inhibitor of the oomycete enzyme, and modifications would be needed to achieve a nanomolar IC_50_.

## Discussion

Oomycetes had been traditionally misclassified as fungi, since they have fungus-like growth morphology, most sharing filamentous growth habits, producing spores and occupying similar environments (Latijnhouwers et al., 2003). However, morphological features, biochemical analyses and molecular taxonomy studies indicate that oomycetes reside more closely to algae and diatoms in the Stramenopile group, and are a sister group to apicomplexa, ciliates, dinoflagellates, and amoeboids in the protists’ supergroup SAR (Adl et al., 2012). The close relation between oomycetes and apicomplexans (Haldar et al., 2006) led us to hypothesize that strategies that have demonstrated effectiveness for control of the human parasites *T. gondii* and *P. falciparum* could also be used to control the growth of *P. infestans*. Indeed, the reverse strategy, the use of oomyceticidal agrochemicals to control apicomplexan parasites, appears to be effective for strobilurins, such as azoxystrobin, trifloxystrobin, and dimoxystrobin, which inhibit *P. falciparum* with nanomolar IC_50_s (Witschel et al., 2012). *De novo* pyrimidine biosynthesis is known to be indispensable for *Toxoplasma gondii* (Fox and Bzik, 2002, 2010) and for more than a decade, the fourth enzyme in the pathway, DHODH, has been studied as a drug target to control malaria (Baldwin et al., 2002; Ashley, 2017). Thus, we decided to characterize these enzymes in the plant pathogen and its host.

As is the case for many organisms, *P. infestans* also possesses a salvage pathway for recycling nucleosides and nucleobases, that together with *de novo* biosynthesis, allow the pathogen to maintain the pyrimidine requirements needed for survival (Garcia-Bayona et al., 2014). Early biotrophic growth is fueled by the *de novo* pathway (Garcia-Bayona et al., 2014), and is also supported by the uptake of pyrimidines from the host environment, as demonstrated by apparent increases in expression of the pathogen’s nucleoside and nucleobase transporters during plant infection (Abrahamian et al., 2016). The fungal plant pathogen *Magnaporthe oryzae* exhibits a similar requirement for *de novo* synthesis for its early biotrophic growth in the case of purine nucleotides, which apparently are not sufficiently available from the plant host (Fernandez et al., 2013). In the solanaceous hosts of *Phytophthora*, such as potato, the *de novo* pathway is important in growing and developing tissues (Giermann et al., 2002). However, studies of knockdowns of pyrimidine biosynthetic enzymes in transgenic plants demonstrate that the salvage pathway can maintain the required pyrimidine pool for basic metabolism (Schroder et al., 2005) and both pathways are tuned in mutually compensatory fluxes (Zrenner et al., 2006). An additional catabolic pathway, not present in the pathogen, allows for fine-tuning of the plant pyrimidine levels (Zrenner et al., 2009).

Dihydroorotate dehydrogenase catalyze the oxidation of dihydroorotate to orotate, and are classified as follows. Class 1 enzymes are soluble, using either fumarate (class 1A) or NAD^+^ (class 1B) as their electron acceptors, and class 2 enzymes are membrane-associated, using ubiquinones in the electron transport chain (ETC) as electron acceptors (Reis et al., 2017). The DHODHs from both *S. tuberosum* and *P. infestans* are class 2 enzymes. The mitochondrial localization of plant DHODHs was demonstrated many years ago. In tomato, DHODH co-sediments with the mitochondrial proteins cytochrome oxidase, succinate dehydrogenase, and citrates synthase in sucrose gradients (Miersch et al., 1986). In pea, DHODH is associated with purified mitochondria, and has been shown to reduce cytochrome c in mitochondrial extracts when cytochrome oxidase is inhibited by cyanide. The N-terminus of a class 2 enzyme contains a mitochondrial targeting sequences followed by hydrophobic transmembrane domain that anchors the enzyme to the inner mitochondrial membrane (Chen and Jones, 1976; Zameitat et al., 2007) The structures available for enzymes from this class show that they are comprised of a large α/β-barrel domain containing the orotate/dihydroorotate and FMN binding sites, connected to a small domain containing two alpha helices, αA and αB, forming a hydrophobic tunnel where ubiquinone is thought to bind (Reis et al., 2017).

Differences were observed in the apparent kinetic parameters of the plant and pathogen DHODHs. The K_m_^app^ for dihydroorotate was threefold higher for ΔN54PiDHODH compared to that of ΔN69StDHODH. In contrast, the K_m_^app^ for the electron acceptor Qd was twofold higher for ΔN69StDHODH compared to that of ΔN54PiDHODH (Table 1). The k_cat_^app^ of the pathogen enzyme was approximately double that of the plant enzyme (Table 1). We tested the activities of the recombinant enzymes with ubiquinones having different isoprenoid tail lengths. While humans use Q10, a ubiquinone with 10 isoprenoid units, both Q9 and Q10 have been found in plants. Ubiquinone content for a few solanaceous plants are available. Tobacco contains Q10 at ≈8 mg/kg (Ohara et al., 2004). Q10 is also found in potato and tomato, but at approximately 10-fold lower concentrations, and levels of Q9 are below the detection limit (Mattila and Kumpulainen, 2001). Although the nature of the quinone in *P. infestans* is not known, the ubiquinone pool in *Phytophthora cactorum* appears to be comprised of 80% Q9, and 20% Q8 (Richards and Hemming, 1972), in agreement with a later study showing that Q9 is the major quinone in other oomycetes (Nakamura et al., 1995). Surprisingly, Q9 was not the best substrate for ΔN54PiDHODH, which exhibited approximately double the activity with ubiquinones having shorter isoprenoid tails. Similarly, ΔN69StDHODH appeared to be more active with ubiquinones having isoprenoid tails shorter than Q10 (Table 2). We also tested the activity of the recombinant enzymes with several 1,4-naphthoquinones (Table 2). These compounds, which affect diverse cellular targets because of their redox properties (Klotz et al., 2014), are alternate electron acceptors for DHODHs (Knecht et al., 2000). For ΔN69StDHODH the best acceptor was 5,8-dihydroxy-1,4-naphthoquinone, which exhibited an activity similar to Qd. In contrast, this naphthoquinone was a poor acceptor for ΔN54PiDHODH, showing sixfold less activity compared to Qd. Differences in activities for the two enzymes were also observed for lawsone, juglone, and plumbagin.

We tested compounds known to inhibit DHODHs from other organisms (Table 3). Alloxan and 5′-fluoroorotic acid, analogs of dihydroorotate and orotate, respectively, are weak inhibitors of other class 2 DHODHs (Zameitat et al., 2006; Munier-Lehmann et al., 2013), and were also poor inhibitors of ΔN69StDHODH and ΔN54PiDHODH. The remaining compounds in Table 3 have been shown to interact, or are predicted to interact, with the ubiquinone binding site, which is the target for the most effective inhibitors known to date of class 2 DHODHs (Munier-Lehmann et al., 2013). The variability in this site in class 2 DHODHs has permitted the development of species-specific inhibitors, as illustrated by DSM-265, a triazolopyrimidine inhibitor of *P. falciparum* DHODH (Ashley, 2017; McCarthy et al., 2017). We found that the related compound DSM190 (Gujjar et al., 2011), had similar and moderate effects on the activities of both recombinant plant and pathogen DHODHs, as did two nanomolar inhibitors of the human enzyme, redoxal (Knecht and Löffler, 2000) and dichloroallyl lawsone (Knecht et al., 2000). Brequinar, another nanomolar inhibitor of the human enzyme (Munier-Lehmann et al., 2013), caused moderate inhibition of ΔN69StDHODH, but did not decrease the activity of ΔN54PiDHODH. Greater inhibition of ΔN69StDHODH compared to ΔN54PiDHODH was also observed for toltrazuril, a weak inhibitor of *Eimeria* pyrimidine biosynthesis (Zameitat et al., 2006; Munier-Lehmann et al., 2013), NSC 61890, and NSC 71097. Several compounds caused greater inhibition of the *Phytophthora* enzyme than the plant enzyme. For example, the active metabolite of leflunomide, A77 1726, a nanomolar HsDHODH inhibitor used in the treatment rheumatoid arthritis, inhibited ΔN54PiDHODH, but was a poor inhibitor of ΔN69StDHODH. The differential effects of modifying a lead compound scaffold on the inhibition of an enzyme target is nicely illustrated by the MD compounds, a series of inhibitors that were designed in attempt to optimize the binding of A77 1726 to the active sites of PfDHODH or HsDHODH (Leban et al., 2005; Davies et al., 2009). While MD 129 did not affect either enzyme’s activity, MD 209 strongly inhibited both enzymes, and MD108 and MD241 preferentially inhibited the pathogen enzyme.

Since we had limited quantities of MD108 and MD241, we selected A77 1726 for further investigation. Measurement of IC_50_s revealed that the pathogen enzyme, with an IC_50_ of 79 μM, was 37-fold more sensitive than the plant enzyme. We found that a concentration of 0.5 mM A77 1726 reduced the growth of the oomycete by approximately 50%, at 92 h. Despite the number of DHODH inhibitor sets available (Munier-Lehmann et al., 2013), only two lipophilic inhibitors have been described and suggested as lead compounds for development of oomyceticides that target this enzyme (Parker et al., 2002). These compounds arrested *Py. aphinadermatum* radial growth *in vitro* and the addition of uridine was able to reverse this growth inhibition, suggesting that the compounds block pyrimidine metabolism. Nevertheless, these compounds were not stable enough under field conditions to be further developed as oomyceticides.

While the role played by the DHODH in *de novo* pyrimidine synthesis is well-defined, it is less clear what role it plays in the respiratory chain of *P. infestans*. In *T. gondii*, the mitochondrially-associated DHODH has an unknown, but essential, pyrimidine-independent function (Hortua Triana et al., 2016). In mammalian cells, physical associations between DHODH and complexes II and III have been demonstrated, and DHODH knockdown partially inhibits complex III, decreases membrane potential, and increases production of reactive oxygen species (Fang et al., 2013). Relatively little is known about the respiratory chains of plant pathogens. In *P. infestans*, inhibition of oxygen consumption is observed in the presence of cyanide and in the presence of antimycin A, when succinate is used as the electron donor (Scheepens and Fehrmann, 1977). Electron transport is reported to be insensitive to rotenone (Scheepens and Fehrmann, 1977). *P. infestans* possesses sequences with similarity to type II NADH dehydrogenases (Feng et al., 2012), plant alternate NADH dehydrogenases (Schertl and Braun, 2014), electron transfer flavoprotein ubiquinone oxidoreductase, and several so-called alternative oxidases (AOX) (Supplementary Table 3). Thus, *P. infestans* mitochondrial respiration appears to exhibit a complexity due to the increased number of branches, as is also observed in plants (Schertl and Braun, 2014). Oomycete respiratory chains are the target of several commercial oomyceticides, ametoctradin (Initium^®^), amisulbrom, and cyazofamid, bind to ubiquinone sites of respiratory complex III, and are effective against late blight disease (Mitani et al., 2001; Dreinert et al., 2018). Since complex III and DHODH both bind ubiquinone, we tested ametoctradin on the recombinant DHODs, and found it had a negligible effect on the plant enzyme, and showed a weak inhibition of the pathogen enzyme. Atovaquone, an antimicrobial ubiquinone analog that inhibits the complexes III of *Plasmodium, T. gondii*, and *Pneumocystis carinii* (Meshnick et al., 2001; Kessl et al., 2003; Mather et al., 2005) likewise showed little effect on either recombinant enzyme.

Taken together, our results highlight differences in binding of DHODH inhibitors by the plant and oomycete enzymes, and suggest that these differences could be further exploited to develop species-specific inhibitors that preferentially affect the pathogen enzyme. The inactivation of this enzyme by inhibitors would have a profound effect on pyrimidine biosynthesis, and might also have effects on the mitochondrial respiration of this pathogen. The availability of recombinant ΔN54PiDHODH should expedite the discovery of more potent agents for growth control strategies against *P. infestans*, and permit the screening of a large number of compounds, the examination of structure-activity relationships of inhibitors, and determination of the 3D structure of enzyme–inhibitor complexes.

## Conclusion

To our knowledge this is first preliminary characterization of a purified recombinant oomycete DHODH and the first comparison with the corresponding plant DHODH. Whether the differences observed between the two enzymes could be further exploited to develop species-specific compounds for crop management is an intriguing question that remains to be answered. The availability and characterization of the recombinant DHODH from *P. infestans* in this work permitted the first preliminary screening of potential enzyme inhibitors, with the rationale of interfering with pyrimidine metabolism, and opens the way to protein crystallization, which is a prerequisite for the development of species-specific inhibitors.

## Author Contributions

MG, HN-O, DP, ML, HJ, SR, and BZ conceived and designed the experiments. MG, HN-O, and DP conducted the experiments. MG, HN-O, DP, and BZ analyzed the data. ML, HJ, SR, and BZ contributed with reagents, materials, and analysis tools. MG, HN-O, and BZ contributed to the writing of the manuscript. All authors reviewed the results and approved the final version of the manuscript.

## Conflict of Interest Statement

The authors declare that the research was conducted in the absence of any commercial or financial relationships that could be construed as a potential conflict of interest. The reviewer WB declared a shared affiliation, with no collaboration, with one of the authors, ML, to the handling Editor at the time of review.

## Footnotes

1 https://plants.ensembl.org

2 http://www.bioinformatics.org/strap/

3 www.graphpad.com

## References

[B1] Abrahamian M. Ah-Fong A. M. V. Davis C. Andreeva K. Judelson H. S. (2016). Gene expression and silencing studies in *Phytophthora infestans* reveal infection-specific nutrient transporters and a role for the nitrate reductase pathway in plant pathogenesis. *PLoS Pathog.* 12:e1006097. 10.1371/journal.ppat.1006097 27936244 PMC5176271

[B2] Adl S. M. Simpson A. G. B. Lane C. E. Lukeš J. Bass D. Bowser S. S. (2012). The revised classification of eukaryotes. *J. Eukaryot. Microbiol.* 59 429–514. 10.1111/j.1550-7408.2012.00644.x 23020233 PMC3483872

[B3] Ah-Fong A. M. V. Judelson H. S. (2011). Vectors for fluorescent protein tagging in *Phytophthora*: tools for functional genomics and cell biology. *Fungal Biol.* 115 882–890. 10.1016/j.funbio.2011.07.001 21872185

[B4] Ashley E. (2017). Investment in antimalarial drug development is bearing fruit. *Lancet. Infect. Dis.* 17 568–570. 10.1016/S1473-3099(17)30172-X 28363638

[B5] Attard A. Gourgues M. Galiana E. Panabières F. Ponchet M. Keller H. (2008). Strategies of attack and defense in plant-oomycete interactions, accentuated for *Phytophthora parasitica* dastur (syn. P. Nicotianae Breda de Haan). *J. Plant Physiol.* 165 83–94. 10.1016/j.jplph.2007.06.011 17766006

[B6] Baldwin J. Farajallah A. M. Malmquist N. A. Rathod P. K. Phillips M. A. (2002). Malarial dihydroorotate dehydrogenase. substrate and inhibitor specificity. *J. Biol. Chem.* 277 41827–41834. 10.1074/jbc.m206854200 12189151

[B7] Baldwin J. Michnoff C. H. Malmquist N. A. White J. Roth M. G. Rathod P. K. (2005). High-throughput screening for potent and selective inhibitors of *Plasmodium falciparum* dihydroorotate dehydrogenase. *J. Biol. Chem.* 280 21847–21853. 10.1074/jbc.M501100200 15795226

[B8] Björnberg O. Grüner A.-C. Roepstorff P. Jensen K. F. (1999). The activity of *Escherichia coli* dihydroorotate dehydrogenase is dependent on a conserved loop identified by sequence homology. mutagenesis, and limited proteolysis. *Biochemistry* 38 2899–2908. 10.1021/bi982352c 10074342

[B9] Chen J. J. Jones M. E. (1976). The cellular location of dihydroorotate dehydrogenase: relation to de novo biosynthesis of pyrimidines. *Arch. Biochem. Biophys.* 176 82–90. 10.1016/0003-9861(76)90143-0 184741

[B10] Claros M. G. Vincens P. (1996). Computational method to predict mitochondrially imported proteins and their targeting sequences. *Eur. J. Biochem.* 241 779–786. 10.1111/j.1432-1033.1996.00779.x 8944766

[B11] Davies M. Heikkila T. McConkey G. A. Fishwick C. W. Parsons M. R. Johnson A. P. (2009). Structure-based design, synthesis, and characterization of inhibitors of human and *Plasmodium falciparum* dihydroorotate dehydrogenases. *J. Med. Chem.* 52 2683–2693. 10.1021/jm800963t 19351152

[B12] Deng X. Gujjar R. El Mazouni F. Kaminsky W. Malmquist N. A. Goldsmith E. J. (2009). Structural plasticity of malaria dihydroorotate dehydrogenase allows selective binding of diverse chemical scaffolds. *J. Biol. Chem.* 284 26999–27009. 10.1074/jbc.M109.028589 19640844 PMC2785385

[B13] Dreinert A. Wolf A. Mentzel T. Meunier B. Fehr M. (2018). The cytochrome bc1 complex inhibitor ametoctradin has an unusual binding mode. *Biochim. Biophys. Acta Bioenerg.* 1859 567–576. 10.1016/j.bbabio.2018.04.008 29704498

[B14] Emanuelsson O. Nielsen H. Brunak S. von Heijne G. (2000). Predicting subcellular localization of proteins based on their N-terminal amino acid sequence. *J. Mol. Biol.* 300 1005–1016. 10.1006/jmbi.2000.3903 10891285

[B15] Faissner S. Gold R. (2018). Oral therapies for multiple sclerosis. *Cold Spring Harb. Perspect. Med.* 9 a032011. 10.1101/cshperspect.a032011 29500302 PMC6314072

[B16] Fang J. Uchiumi T. Yagi M. Matsumoto S. Amamoto R. Takazaki S. (2013). Dihydro-orotate dehydrogenase is physically associated with the respiratory complex and its loss leads to mitochondrial dysfunction. *Biosci. Rep.* 33:e00021. 10.1042/BSR20120097 23216091 PMC3564035

[B17] Feng Y. Li W. Li J. Wang J. Ge J. Xu D. (2012). Structural insight into the type-II mitochondrial NADH dehydrogenases. *Nature* 491 478–482. 10.1038/nature11541 23086143

[B18] Fernandez J. Yang K. T. Cornwell K. M. Wright J. D. Wilson R. A. (2013). Growth in rice cells requires de novo purine biosynthesis by the blast fungus *Magnaporthe oryzae*. *Sci. Rep.* 3:2398. 10.1038/srep02398 23928947 PMC3738970

[B19] Fox B. A. Bzik D. J. (2002). De novo pyrimidine biosynthesis is required for virulence of Toxoplasma gondii. *Nature* 415 926–929. 10.1038/415926a 11859373

[B20] Fox B. A. Bzik D. J. (2010). Avirulent uracil auxotrophs based on disruption of orotidine-5’-monophosphate decarboxylase elicit protective immunity to *Toxoplasma gondii*. *Infect. Immun.* 78 3744–3752. 10.1128/IAI.00287-210 20605980 PMC2937452

[B21] Fragoso Y. D. Brooks J. B. B. (2015). Leflunomide and teriflunomide: altering the metabolism of pyrimidines for the treatment of autoimmune diseases. *Expert Rev. Clin. Pharmacol.* 8 315–320. 10.1586/17512433.2015.1019343 25712857

[B22] Fry W. (2008). *Phytophthora* infestans: the plant (and R gene) destroyer. *Mol. Plant Pathol.* 9 385–402. 10.1111/j.1364-3703.2007.00465.x 18705878 PMC6640234

[B23] Fukasawa Y. Tsuji J. Fu S.-C. Tomii K. Horton P. Imai K. (2015). MitoFates: improved prediction of mitochondrial targeting sequences and their cleavage sites. *Mol. Cell. Proteomics* 14 1113–1126. 10.1074/mcp.M114.043083 25670805 PMC4390256

[B24] Garcia-Bayona L. Garavito M. F. Lozano G. L. Vasquez J. J. Myers K. Fry W. E. (2014). De novo pyrimidine biosynthesis in the oomycete plant pathogen *Phytophthora* infestans. *Gene* 537 312–321. 10.1016/j.gene.2013.12.009 24361203

[B25] Giermann N. Schroder M. Ritter T. Zrenner R. (2002). Molecular analysis of de novo pyrimidine synthesis in solanaceous species. *Plant Mol. Biol.* 50 393–403. 12369616 10.1023/a:1019854531254

[B26] Goodwin S. B. Smart C. D. Sandrock R. W. Deahl K. L. Punja Z. K. Fry W. E. (1998). Genetic change within populations of *Phytophthora* infestans in the United States and Canada during 1994 to 1996: role of migration and recombination. *Phytopathology* 88 939–949. 10.1094/PHYTO.1998.88.9.939 18944872

[B27] Gujjar R. El Mazouni F. White K. L. White J. Creason S. Shackleford D. M. (2011). Lead optimization of aryl and aralkyl amine-based triazolopyrimidine inhibitors of *Plasmodium falciparum* dihydroorotate dehydrogenase with antimalarial activity in mice. *J. Med. Chem.* 54 3935–3949. 10.1021/jm200265b 21517059 PMC3124361

[B28] Gullino M. L. Tinivella F. Garibaldi A. Kemmitt G. M. Bacci L. Sheppard B. (2010). Mancozeb, past, present, and future. *Plant Dis.* 94 1076–1087. 10.1094/PDIS-94-9-1076 30743728

[B29] Haldar K. Kamoun S. Hiller N. L. Bhattacharje S. van Ooij C. (2006). Common infection strategies of pathogenic eukaryotes. *Nat. Rev. Microbiol.* 4 922–931. 10.1038/nrmicro1549 17088934

[B30] Heikkilä T. Thirumalairajan S. Davies M. Parsons M. R. McConkey A. G. Fishwick C. W. G. (2006). The first de novo designed inhibitors of Plasmodium falciparum dihydroorotate dehydrogenase. *Bioorg. Med. Chem. Lett.* 16 88–92. 10.1016/j.bmcl.2005.09.045 16236496

[B31] Henniger H. (2007). Zur Kultur von *Phytophthora* infestans auf vollsynthetischen Nährsubstraten. *Z. Allg. Mikrobiol.* 3 126–135. 10.1002/jobm.19630030204

[B32] Hortua Triana M. A. Cajiao Herrera D. Zimmermann B. H. Fox B. A. Bzik D. J. (2016). Pyrimidine pathway-dependent and -independent functions of the Toxoplasma gondii mitochondrial dihydroorotate dehydrogenase. *Infect. Immun.* 84 2974–2981. 10.1128/IAI.00187-116 27481247 PMC5038078

[B33] Hortua Triana M. A. Huynh M.-H. Garavito M. F. Fox B. A. Bzik D. J. Carruthers V. B. (2012). Biochemical and molecular characterization of the pyrimidine biosynthetic enzyme dihydroorotate dehydrogenase from Toxoplasma gondii. *Mol. Biochem. Parasitol.* 184 71–81. 10.1016/j.molbiopara.2012.04.009 22580100 PMC3855825

[B34] Hurt D. E. Widom J. Clardy J. (2006). Structure of *Plasmodium falciparum* dihydroorotate dehydrogenase with a bound inhibitor. *Acta Crystallogr. Sect. D Biol. Crystallogr.* 62 312–323. 10.1107/S0907444905042642 16510978

[B35] Judelson H. S. Spielman L. J. Shattock R. C. (1995). Genetic mapping and non-Mendelian segregation of mating type loci in the oomycete. *Phytophthora infestans*. *Genetics* 141 503–512. 8647388 10.1093/genetics/141.2.503 PMC1206751

[B36] Judelson H. S. Tyler B. M. Michelmore R. W. (1991). Transformation of the oomycete pathogen. *Phytophthora infestans*. *Mol. Plant. Microbe. Interact.* 4 602–607. 1804404 10.1094/mpmi-4-602

[B37] Kessl J. J. Lange B. B. Merbitz-Zahradnik T. Zwicker K. Hill P. Meunier B. (2003). Molecular basis for atovaquone binding to the cytochrome bc 1 complex. *J. Biol. Chem.* 278 31312–31318. 10.1074/jbc.M304042200 12791689

[B38] Klotz L.-O. Hou X. Jacob C. (2014). 1,4-Naphthoquinones: from oxidative damage to cellular and inter-cellular signaling. *Molecules* 19 14902–14918. 10.3390/molecules190914902 25232709 PMC6270801

[B39] Knecht W. Henseling J. Löffler M. (2000). Kinetics of inhibition of human and rat dihydroorotate dehydrogenase by atovaquone, lawsone derivatives, brequinar sodium and polyporic acid. *Chem. Biol. Interact* 124 61–76. 10.1016/s0009-2797(99)00144-1 10658902

[B40] Knecht W. Löffler M. (2000). Redoxal as a new lead structure for dihydroorotate dehydrogenase inhibitors: a kinetic study of the inhibition mechanism. *FEBS Lett.* 467 27–30. 10.1016/s0014-5793(00)01117-0 10664450

[B41] Lamour K. H. Win J. Kamoun S. (2007). Oomycete genomics: new insights and future directions. *FEMS Microbiol. Lett.* 274 1–8. 10.1111/j.1574-6968.2007.00786.x 17559387

[B42] Latijnhouwers M. de Wit P. J. Govers F. (2003). Oomycetes and fungi: similar weaponry to attack plants. *Trends Microbiol.* 11 462–469. 10.1016/j.tim.2003.08.002 14557029

[B43] Leban J. Kralik M. Mies J. Gassen M. Tentschert K. Baumgartner R. (2005). SAR, species specificity, and cellular activity of cyclopentene dicarboxylic acid amides as DHODH inhibitors. *Bioorg. Med. Chem. Lett.* 15 4854–4857. 10.1016/j.bmcl.2005.07.053 16143532

[B44] Liu S. Neidhardt E. A. Grossman T. H. Ocain T. Clardy J. (2000). Structures of human dihydroorotate dehydrogenase in complex with antiproliferative agents. *Structure* 8 25–33. 10.1016/s0969-2126(00)00077-0 10673429

[B45] Manrique-Carpintero N. C. Coombs J. J. Pham G. M. Laimbeer F. P. E. Braz G. T. Jiang J. (2018). Genome reduction in tetraploid potato reveals genetic load, haplotype variation, and loci associated with agronomic traits. *Front. Plant Sci.* 9:944. 10.3389/fpls.2018.00944 30018631 PMC6037889

[B46] Mather M. W. Darrouzet E. Valkova-Valchanova M. Cooley J. W. McIntosh M. T. Daldal F. (2005). Uncovering the molecular mode of action of the antimalarial drug atovaquone using a bacterial system. *J. Biol. Chem.* 280 27458–27465. 10.1074/jbc.M502319200 15917236 PMC1421511

[B47] Mattila P. Kumpulainen J. (2001). Coenzymes Q9and Q10: contents in foods and dietary intake. *J. Food Compos. Anal.* 14 409–417. 10.1006/JFCA.2000.0983

[B48] McCarthy J. S. Lotharius J. Rückle T. Chalon S. Phillips M. A. Elliott S. (2017). Safety, tolerability, pharmacokinetics, and activity of the novel long-acting antimalarial DSM265: a two-part first-in-human phase 1a/1b randomised study. *Lancet Infect. Dis.* 17 626–635. 10.1016/S1473-3099(17)30171-30178 28363636 PMC5446412

[B49] Meshnick S. R. Berry E. A. Nett J. Kazanjian P. Trumpower B. (2001). The interaction of atovaquone with the P. carinii cytochrome bc1 complex. *J. Eukaryot. Microbiol.* 48 169S–171S. 10.1111/j.1550-7408.2001.tb00505.x 11906048

[B50] Miersch J. Krauss G.-J. Metzger U. (1986). Properties and subcellular localization of dihydroorotate dehydrogenase in cells of tomato suspension culture. *J. Plant Physiol.* 122 55–66. 10.1016/S0176-1617(86)80084-0

[B51] Mitani S. Araki S. Takii Y. Ohshima T. Matsuo N. Miyoshi H. (2001). The biochemical mode of action of the novel selective fungicide cyazofamid: specific inhibition of mitochondrial complex III in *Phythium spinosum*. *Pestic. Biochem. Physiol.* 71 107–115. 10.1006/PEST.2001.2569

[B52] Munier-Lehmann H. Vidalain P.-O. Tangy F. Janin Y. L. (2013). On dihydroorotate dehydrogenases and their inhibitors and uses. *J. Med. Chem.* 56 3148–3167. 10.1021/jm301848w 23452331

[B53] Nakamura K. Yuasa K. Sinmuk S. Hatai K. Hara N. (1995). The ubiquinone system in Oomycetes. *Mycoscience* 36 121–123. 10.1007/BF02268582

[B54] Nielsen H. Engelbrecht J. Brunak S. von Heijne G. (1997). Identification of prokaryotic and eukaryotic signal peptides and prediction of their cleavage sites. *Protein Eng.* 10 1–6. 10.1093/protein/10.1.1 9051728

[B55] Nørager S. Jensen K. F. Björnberg O. Larsen S. (2002). E. coli dihydroorotate dehydrogenase reveals structural and functional distinctions between different classes of dihydroorotate dehydrogenases. *Structure* 10 1211–1223. 10.1016/s0969-2126(02)00831-6 12220493

[B56] Ohara K. Kokado Y. Yamamoto H. Sato F. Yazaki K. (2004). Engineering of ubiquinone biosynthesis using the yeast coq2 gene confers oxidative stress tolerance in transgenic tobacco. *Plant J.* 40 734–743. 10.1111/j.1365-313X.2004.02246.x 15546356

[B57] Oliver J. D. Sibley G. E. M. Beckmann N. Dobb K. S. Slater M. J. McEntee L. (2016). F901318 represents a novel class of antifungal drug that inhibits dihydroorotate dehydrogenase. *Proc. Natl. Acad. Sci. U.S.A.* 113 12809–12814. 10.1073/pnas.1608304113 27791100 PMC5111691

[B58] Parker M. H. Durst G. L. Hannum A. C. Henry M. J. Lawler L. K. Smith A. J. (2002). The identification and optimization of oomycete dihydroorotate dehydrogenase inhibitors as fungicides. *Synth. Chem. Agrochem.* 800 303–313. 10.1021/bk-2002-0800.ch028

[B59] Rawls J. Knecht W. Diekert K. Lill R. Löffler M. (2000). Requirements for the mitochondrial import and localization of dihydroorotate dehydrogenase. *Eur. J. Biochem.* 267 2079–2087. 10.1046/j.1432-1327.2000.01213.x 10727948

[B60] Reis R. A. G. Calil F. A. Feliciano P. R. Pinheiro M. P. Nonato M. C. (2017). The dihydroorotate dehydrogenases: past and present. *Arch. Biochem. Biophys.* 632 175–191. 10.1016/j.abb.2017.06.019 28666740

[B61] Richards J. B. Hemming F. W. (1972). Dolichols, ubiquinones, geranylgeraniol and farnesol as the major metabolites of mevalonate in *Phytophthora cactorum*. *Biochem. J.* 128 1345–1352. 10.1042/bj1281345 4643705 PMC1174023

[B62] Rodenburg S. Y. A. Seidl M. F. de Ridder D. Govers F. (2018). Genome-wide characterization of *Phytophthora* infestans metabolism: a systems biology approach. *Mol. Plant Pathol.* 19 1403–1413. 10.1111/mpp.12623 28990716 PMC6638193

[B63] Scheepens P. C. Fehrmann H. (1977). Metabolic anomalies as a possible cause of biotrophy: abbreviated electron transport in *Phytophthora* infestans. *Physiol. Plant Pathol.* 11 71–78. 10.1016/0048-4059(77)90089-90083

[B64] Schertl P. Braun H.-P. (2014). Respiratory electron transfer pathways in plant mitochondria. *Front. Plant Sci.* 5:163. 10.3389/fpls.2014.00163 24808901 PMC4010797

[B65] Schroder M. Giermann N. Zrenner R. (2005). Functional analysis of the pyrimidine de novo synthesis pathway in solanaceous species. *Plant Physiol.* 138 1926–1938. 10.1104/pp.105.063693 16024685 PMC1183384

[B66] Sykes D. B. Kfoury Y. S. Mercier F. E. Wawer M. J. Law J. M. Haynes M. K. (2016). Inhibition of dihydroorotate dehydrogenase overcomes differentiation blockade in acute myeloid leukemia. *Cell* 167 171.e–186.e. 10.1016/j.cell.2016.08.057 27641501 PMC7360335

[B67] Tellier F. Fritz R. Kerhoas L. Ducrot P.-H. Einhorn J. Carlin-Sinclair A. (2008). Characterization of metabolites of fungicidal cymoxanil in a sensitive strain of *Botrytis cinerea*. *J. Agric. Food Chem.* 56 8050–8057. 10.1021/jf8010917 18693740

[B68] Tusnady G. E. Simon I. (2001). The HMMTOP transmembrane topology prediction server. *Bioinformatics* 17 849–850. 10.1093/bioinformatics/17.9.849 11590105

[B69] Ullrich A. Knecht W. Fries M. Loffler M. (2001). Recombinant expression of N-terminal truncated mutants of the membrane bound mouse, rat and human flavoenzyme dihydroorotate dehydrogenase. a versatile tool to rate inhibitor effects? *Eur. J. Biochem.* 268 1861–1868. 10.1046/j.1432-1033.2001.02061.x 11248707

[B70] Ullrich A. Knecht W. Piskur J. Loffler M. (2002). Plant dihydroorotate dehydrogenase differs significantly in substrate specificity and inhibition from the animal enzymes. *FEBS Lett.* 529 346–350. 10.1016/s0014-5793(02)03425-7 12372626

[B71] Vargas A. M. Quesada Ocampo L. M. Cespedes M. C. Carreno N. Gonzalez A. Rojas A. (2009). Characterization of *Phytophthora* infestans populations in Colombia: first report of the A2 mating type. *Phytopathology* 99 82–88. 10.1094/PHYTO-99-1-0082 19055438

[B72] Witschel M. Rottmann M. Kaiser M. Brun R. (2012). Agrochemicals against malaria, sleeping sickness, leishmaniasis and chagas disease. *PLoS Negl. Trop. Dis.* 6:e1805. 10.1371/journal.pntd.0001805 23145187 PMC3493374

[B73] Witz S. Jung B. Furst S. Mohlmann T. (2012). De novo pyrimidine nucleotide synthesis mainly occurs outside of plastids, but a previously undiscovered nucleobase importer provides substrates for the essential salvage pathway in *Arabidopsis*. *Plant Cell* 24 1549–1559. 10.1105/tpc.112.096743 22474184 PMC3398563

[B74] Zameitat E. Freymark G. Dietz C. D. Löffler M. Bölker M. (2007). Functional expression of human dihydroorotate dehydrogenase (DHODH) in pyr4 mutants of ustilago maydis allows target validation of DHODH inhibitors in vivo. *Appl. Env. Microbiol.* 73 3371–3379. 10.1128/AEM.02569-2566 17369345 PMC1907109

[B75] Zameitat E. Gojkoviæ Z. Knecht W. Piškur J. Löffler M. (2006). Biochemical characterization of recombinant dihydroorotate dehydrogenase from the opportunistic pathogenic yeast Candida albicans. *FEBS J.* 273 3183–3191. 10.1111/j.1742-4658.2006.05327.x 16774642

[B76] Zrenner R. Riegler H. Marquard C. R. Lange P. R. Geserick C. Bartosz C. E. (2009). A functional analysis of the pyrimidine catabolic pathway in *Arabidopsis*. *New Phytol.* 183 117–132. 10.1111/j.1469-8137.2009.02843.x 19413687 PMC2713857

[B77] Zrenner R. Stitt M. Sonnewald U. Boldt R. (2006). Pyrimidine and purine biosynthesis and degradation in plants. *Annu. Rev. Plant Biol.* 57 805–836. 10.1146/annurev.arplant.57.032905.105421 16669783

