# Supplementary material for: Phytophthora infestans Dihydroorotate Dehydrogenase Is a Potential Target for Chemical Control – A Comparison With the Enzyme From Solanum tuberosum
Source: Front Microbiol. 2019 Jun 28;10:1479. doi: 10.3389/fmicb.2019.01479 (PMC6611227; doi:10.3389/fmicb.2019.01479)
Supplement: Supplementary file 1 [file Data_Sheet_1.PDF]

Supplementary Material

Phytophthora infestans dihydroorotate dehydrogenase is a potential target for chemical control – a comparison with the enzyme from *Solanum tuberosum*.

Manuel F. Garavito[1,2†‡], Heidy Y. Narváez-Ortiz[1,†‡], Dania Camila Pulido[1‡], Monika Löffler[3], Howard S. Judelson[4], Silvia Restrepo[2], Barbara H. Zimmermann[1*].

[1]Departamento de Ciencias Biológicas, Universidad de los Andes, Carrera 1 # 18A – 10, Bogotá D.C., Colombia

[2]Laboratorio de Micología y Fitopatología, Universidad de los Andes, Carrera 1 # 18A – 10, Bogotá D.C., Colombia

[3]Faculty of Medicine, Department of Biology, Philipps University Marburg, Karl-von-Frisch-Str. 8, D-35032 Marburg, Germany

[4]Department of Microbiology and Plant Pathology, University of California, Riverside, CA, USA

† Shared first authorship.

‡ Present address: MFG, Wisconsin Institute for Discovery and Department of Plant Pathology, University of Wisconsin, Madison, WI, USA. HYN-O, Institute of Molecular Biology, University of Oregon, 1229 University of Oregon, Eugene, OR, USA. DCP, Boyce Thompson Institute and Department of Chemistry and Chemical biology, Cornell University, Ithaca, NY, USA.

*Communicating author. bazimmer@uniandes.edu.co

Supplementary Figure 1. Alignment of the predicted amino acid sequences of DHODHs from oomycetes. Predicted electron acceptor binding site (αA and αB), FMN and orotate binding sites, are underlined. *Phytophthora infestans* AFP19903.1 (Pinf); *Phytophthora parasitica* ETN24904.1 (Ppar); *Aphanomyces invadans* ETW02915.1 (Ainv); *Aphanomyces astaci* ETV71768.1 (Aast); *Saprolegnia parasitica* XP_012208225.1 (Spar); *Saprolegnia diclina* EQC33802.1 (Sdic); *Phytophthora sojae* EGZ21201.1 (Pram); *Plasmopara halstedii* CEG35261.1 (Phal) and *Pythium ultimum var. ultimum DAOM BR144* PYU1_T005811 (Pult).

```
Spar     -MFR----AVARPLAA-QS----RRQ---PSARHMSSIPTGSSSPLT---PLLLGVGAG--L-----VYFNRQRLLKSLCDP
Sdic     -MFR----AVARPLAA-VS---RRRQ---PSARHMSSIPTGSGSPLT---PLLLGVGAG--L-----IYFNRQRLLKALCDP
Ainv     -MYRRCMGGVLRRSA--RC---PSAG---GSRRLMSSIPSSSSSSAA---PLILGAGVL--A-----LYTFRQSFLTKLMDP
Aast     --MHRVLNVVARPAARSAL---AMHG---TSRRCMSSIPTGSSSPVG---PILLGATAL--G-----LYTFRQSFLTTFMDP
Pult     MHVLRTLRHSQRQATLASSALTAKRSADVARFSTQSSSPKSSSGGFRFAEMVALAGGIGAAGVAISQGFVPHEWVIRQLSEP
Phal     --------------------MGLTRRTSRKLQTMSFRSTAVSPTGVHATLYLAIGGGAAAAVVAAFKGIVPHEWVIRQLAEP
Psoj     ----------MRAATTS--ICRASRRALAQQAACMSTGPSSSFSGLHTAAYLAIGGGAAAAVVAAANGVVPHEWVIRQLAEP
Pinf     ----------MRAATFS--IRRARRTLQ----TSSFSSAASSSSGFHTAAYVAVGGGAAAAVVAAYNGVVPHEWVIRQLAEP
Ppar     ----------MRAATSS--IRRASRTLQ----SASFSSAASSSSGFHTAAYVAVGGGAAAAVVAAYNGVVPHEWVIRQLAEP
                                                       Transmembrane domain               αA
Spar     VLMPAVRLFDPETAHILAVKAAKYGLIPKDKRHDDPSLQVTAFNLTFDNPLGIAAGFDKHAEAMQGLLDMGFGFVEIGSVTP
Sdic     VLMPAVRLFDPETAHILAVKAAKYGLIPKDKRHDDPSLKVTAFNLTFDNPLGIAAGFDKHAEAMQGLLDMGFGFVEIGSVTP
Ainv     VLMPLLRLLDPETSHVLAVQAAKYGWTPKDTLPDDTSLRMSVLGMSFDNPIGIAAGFDKHADAMQGLLDMGFGFVEIGSVTP
Aast     VLMPLLRLLGPETSHVLAVQAAKYGWSVKDTVPDDPSLHVSLLGLSFDNPIGIAAGFDKHADAMQGLLDMGFGFVEIGSVTP
Pult     VLMPLVRLFDPETAHVIAVKSAALGLIPRDHGTDSLVLSVKAFGQEFPNPLGMAAGFDKNAEAIEGLLDIGFGFVEIGSVTP
Phal     M-MPVIRIFDPETAHNIAVQCARFGLIPKDPEPDPKLLHVHALGLKFSNPLGIAAGFDKDGQAMEGMLDMGFGCVEIGSVTP
Psoj     M-MPVVRLFDPETAHQVAVQCARFGLTPKDPEADPELLRVQALGLEFPNPLGIAAGFDKHGEAMEGMLDMGFGCVEIGSVTP
Pinf     M-MPVVRMFEPETAHKIAVQCARFGLTPKDPETDPELLHVQVLGLEFTNPLGIAAGFDKDGEAMEGMLDMGFGCVEIGSVTP
Ppar     M-MPVVRLFEPETAHKIAVQCARFGLTPKDPETDPELLHVKALGLEFTNPLGIAAGFDKHGEAMEGMLDMGFGCVEIGSVTP
                     αB                                                             FMN binding
Spar     LAQSGNPKPRVFRLTEDRGVINRYGFNSVGADVVQKRLQRYAYWALERPQYASVRAGPLGINLGKNKTSPSTIADYVAGVEK
Sdic     LAQSGNPKPRVFRLTEDRGVINRYGFNSVGADAVQKRLQRYAYWALERPQYASVRAGPLGINLGKNKTSPSTIADYVAGVEK
Ainv     LPQEGNPKPRVFRLLEDRGVINRYGFNSEGHPKVRERLEKYKYWTLSSSTKLHHRTGPLGVNLGKNKTSDSPIDDYVRGVES
Aast     LPQDGNPKPRVFRLVEDRGVINRYGFNSQGHAKVRERLEKYKYWTLSTTTSKQYRRGPLGVNLGKNKSSDSPIEDYVRGVET
Pult     KPQPGNPKPRVFRLTEDRGVINRYGFNSSGMDAVAKSLESYVTTRE-L-SKRFHRSGVLGVNLGKNKTTEDAAADYVEGVYT
Phal     KPQPGNAKPRVFRLPEDRGVINRYGFNSKGLEHVSTRLEKYVSSRA-KRQKAGHRAGILGVNLGKNKLTEDAAADYVQGVHA
Psoj     KPQPGNPQPRVFRLPEDRGVINRYGFNSKGLEYVGDRLERYVGSRA-KRQGNGHRAGVLGVNLGKNKLTEDAASDYVRGVHA
Pinf     KAQPGNPTPRVFRLPEDRGVINRYGFNSKGLEHVGARLERYVSSRV-NRQDDGHRAGVLGVNLGKNKLTEDAAADYVQGVHA
Ppar     KPQPGNPTPRVFRLAEDRGVINRYGFNSKGLEYVGARLERYVGSRA-KRQENGHRAGVLGVNLGKNKLTEDAAADYVQGVHA
         site
Spar     LGPYGDYLVINISSPNTPGLRSLQGKQELEALVAAVLEARNKL----WKRLPLLVKIAPDLTLEDQKDIADVALALQIDGLI
Sdic     LGPYGDYLVINISSPNTPGLRSLQGKQELEALVAAVLEARNKL----WKRLPLLVKIAPDLTPEDQKDIADVALALQIDGLI
Ainv     LGPFGDYLVINISSPNTPGLRSLQGKKELHALVSAVLDARNKL----WKRVPLLVKIAPDLTPEDMQDIADVVVALKVDGLI
Aast     LGPFGDYLVINISSPNTPGLRSLQGKKELHALVSAVLDARNKL----WKRLPLLVKIAPDLTSDDMRDIAEVALALQIDGLI
Pult     LGKYADYLVVNVSSPNTPGLRSLQGKQQLQELLTRVLDARKNVEAKENRKIPLLVKIAPDLTEDDKKDIADVAVELNLDGLI
Phal     LGKFADYLVVNVSSPNTPGLRTLQGKQQLQNLLVRVLKARNEVAAIEKRNIPLLVKIAPDLTEDDKQDIADVALELKLDGLV
Psoj     LGKYADYLVVNVSSPNTPGLRTLQGKIQLQNLLVRVLKARDEVAAKEERRIPLLVKIAPDLTEDDKQDIADVALELKLDGLV
Pinf     LGKYADYLVVNVSSPNTPGLRTLQGKIQLQKLLEHVLKARDEVADTEKRTISLLVKIAPDLTEDDKQDIADVALALKLDGLV
Ppar     LGKYADYLVVNVSSPNTPGLRTLQGKIQLQKLLERVLKARDEVAATEKRNIPLLKIAPDLTEDDKQDIADVALELKLDGLVV
              Orotate binding site
Spar     VSNTTISRPESLHSEHKGETGGLSGAPVKDISTKVLHSMYALTQGKIPLIGVGGVATGQDAYEKIRAGATLVQMYSCLVFDG
Sdic     VSNTTISRPESLTSEHKGETGGLSGAPVKDISTKVLHSMYALTHGKIPLIGVGGVATGQDAYEKIRAGATLVQMYSCLVYDG
Ainv     VSNTTISRPPSLTSPHAAETGGLSGAPVKELSTAVLSSMYKLTQGKIPLIGVGGVATGHDAYDKIKAGASLVQLYSSLVFDG
Aast     VSNTTISRPESLLSPHAAETGGLSGAPVKELSTTVLHSMYKLTEGKIPLIGVGGVATGQDAYDKIRAGASLVQLYSSLVFNG
Pult     VSNTTLSRPDTLKSSHKIETGGLSGAPVRDLSTQVLSDMYKLTNGMIPLIGVGGVSSGKDAYEKICAGATLVQMYSCMVYDG
Phal     VSNTTLRRPDTLKGAAKTEIGGLSGLPVRELSTKVLGDMYKLTKGKITLIGVGGVSSGQDAYDKIRAGASLVQMYSCLIYES
Psoj     VSNTTLSRPDTLKGEAKGETGGLSGLPVRDLSTKVLGDMYKLTKGQIPLIGVGGVSTGQDAYDKIRAGASLVQMYSCLIYES
Pinf     VSNTTLSRPDTLKGAAKGETGGLSGFPVRDMSTKVLGDMYKLTKGKIPLIGVGGVSTGQDAYDKIRAGASLVQMYSCLIYES
Ppar     VSNTTLSRPDTLKGAAKGETGGLSGLPVRDLSTKVLGDMYKLTKGQIPLIGVGGVSTGQDAYDKIRAGASLVQMYSCLIYES

Spar     PMAVPRIKEELAACLQRDGFASVHDAIGAAHAPTE--------
Sdic     PMAVPRIKEELAACLQRDGFASVHDAIGAAHKST---------
Ainv     PLAVARIKHELTALIKADGYTSVAEAVGAAHRVLPATKTAPPA
Aast     PLAVARIKHELTACIKQDGYTSVAEAVGAAHNDPSSKNPQP—
Pult     PTAVPRAKQELEALLLADGYKNVVDAIGAAHKK----------
Phal     PLAIPRAKKELASLLQRDGYESVADAVGAAHK-----------
Psoj     PLAVPRAKKELVALLRRDGYESVADAVGAAHK-----------
Pinf     PLAVPRAKKELAALLSRDGYKSVIDAVGAAHK-----------
Ppar     PLAVPRAKKELAALLSRDGYTSVTEAVGTAHK-----------
```

Supplementary Figure 2. Denaturing gel electrophoresis of purified recombinant truncated DHODHs from *Solanum tuberosum* (St, ΔN69StDHODH) and *Phytophthora infestans* (Pi, ΔN54PiDHODH). Both proteins were expressed in *Escherichia coli* BL21-CodonPlus(DE3)-RP with N-terminal His-tags, and were purified using Co^{2+} affinity columns. Bands are observed near the expected molecular masses of 43.7 kDa (St), and 43.1 kDa (Pi).

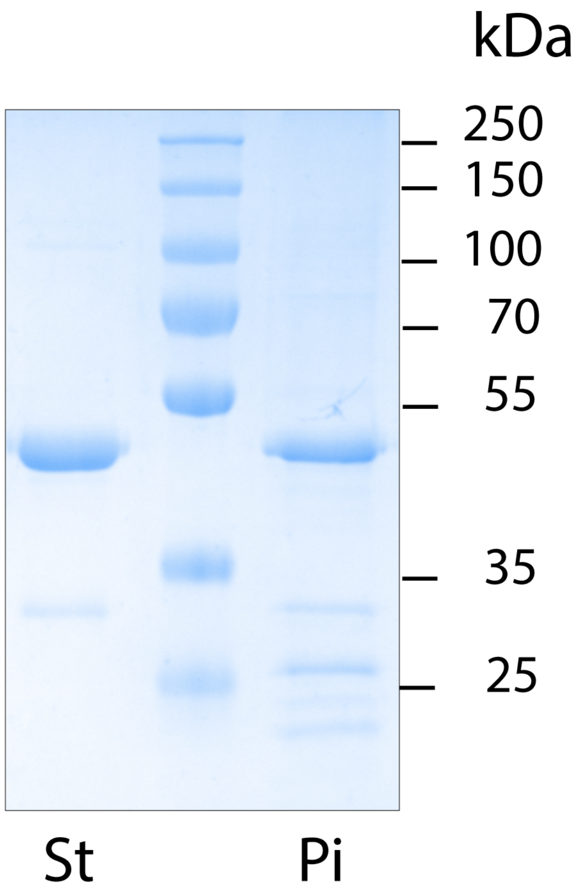

Supplementary Table 1. List of primers used in this study.

	Organism	Construct	Plasmid	Forward (5`-3`)	Reverse (5`-3`)	Length (bp)	Length (aa)
**Activity**	*Pi*	PiDHOD	pET19b	caggatcctATGCGAGCAGCGACGTTCTC	caggatccTTACTTGTGAGCAGCGCCTACG	1275	424
	Pi	ΔN69StDHODH	pET19b	caggatcctCCGCATGAGTGGGTCATC	caggatccTTACTTGTGAGCAGCGCCTACG	1113	370
	St	ΔN54PiDHODH	pET15b	tatcggcatatgGCAAGTACAGTTGATGAGGCC	ccctcgagTCATGCTCCAACAGCTTC	1134	378
**Localization**	**Construct**		**Plasmid**	**Forward (5`-3`)**	**Reverse (5`-3`)**	**Length (bp)**	**Length (aa)**
	PiDHODH + GFP		pGFPH	gggTTAATTAAtATGCGAGCAGCGACGTTC	gggGCTAGCCTTGTGAGCAGCGCCTACG	1272+744	424+247
**Sequencing (localization)**	**Sequence amplified**			**Forward (5`-3`)**	**Reverse (5`-3`)**		
	HAMpromoter-HAMterminator			ACTTCCTCTTTTACATCCGA	ATCACCGATTCGTCACAC		
	HAMpromoter-GFPreporter				CACGGAACAGGGAGCTTT		

Sequences added to facilitate cloning are indicated in lower case.

Supplementary Table 2.
Percent amino acid sequence identity of DHODs from different organisms.

	Ecol	Hsap	Pfal	Pinf	Stub	Zmay	Atha	Scer
**Ecol**								
**Hsap**	40							
**Pfal**	39	36						
**Pinf**	38	53	31					
**Stub**	41	49	30	48				
**Zmay**	39	52	30	50	73			
**Atha**	39	48	29	46	77	73		
**Scer**	19	19	20	19	21	21	21	
**Tcru**	18	22	19	22	21	24	23	53

Abbreviations and accession numbers are: Ecol, *E. coli*, 1F76 – WP_001295934; Hsap, *Homo sapiens*, 1D3G – NP_001352; Pfal, *P. falciparum*, 1TV5 – XP_966023; Pinf, *P. infestans*, PITG_01913; Stub, *S. tuberosum*, PGSC0003DMG401016396; Zmay, Zea mays, NP_001152058; Atha, *A. thaliana*, AAN64025; Scer, *Saccharomyces cerevisiae*, CAA42014; Tcru, *Trypanosoma cruzi*, BAE48283. Multiple sequence alignments were performed according to sequence and available 3D-structure (for Ecol, Hsap, Pfal, PDB codes are indicated prior to the sequence accession number) using the STRAP program (http://www.bioinformatics.org/strap/).

Supplementary Table 3. Selected enzymes involved in the electron transport of the inner mitochondrial membrane of *A. thaliana* and related proteins of *P. infestans.*

Enzyme or Complex[1]	Abbreviation		Accession *P. infestans*	Accession *A. thaliana*	% identity (% coverage)
Dihydroorotate dehydrogenase	DHODH		PITG_01913	At5g23300	54 (80)
Glycerol-3-phosphate dehydrogenase	GDPH		PITG_06719	At3g10370	43 (97)
Electron transfer flavoprotein-ubiquinone oxidoreductase	ETFQO		PITG_02092	At2g43400	55 (89)
Electron transfer flavoprotein α	ETF α		PITG_12882	At1g50940	55 (89)
Electron transfer flavoprotein β	ETF β		PITG_10300	At5g43430	58 (89)
Alternative NAD(P)H dehydrogenase 1 (internal)	NDA1<br>NDA2		PITG_14863[2]	At1g07180<br>At2g29990	58 (89)<br>35 (86)
NAD(P)H dehydrogenase C (internal)	NDC1		–	At5g08740	-
NAD(P)H dehydrogenase B (external)	NDB1<br>NDB2[3]<br>NDB3[3]<br>NDB4[3]		PITG_14863[2]	At4g28220<br>At4g05020<br>At4g21490<br>At2g20800	34 (78)<br>34 (73)<br>35 (74)<br>36 (73)
Complex I[4]	NUAM	75 kDa subunit	–	At5g37510	-
	NUHM	24 kDa subunit	PITG_13683	At4g02580	51 (72)
	NUBM	51 kDa subunit	PITG_06815	At5g08530	71 (94)
	NUCM	connecting domain (rotenone)[5]	–	AtMg00510	-
	NUGM	connecting domain	AAW67078	AtMg00070	54 (84)
	NUIM	connecting domain	PITG_14612	At1g79010	63 (95)
	NUKM	connecting domain	PITG_09031<br>PITG_09015	At5g11770	60 (93)
	NU1M	membrane domain	ACT75638	AtMg00516<br>AtMg01120<br>AtMg01275<br>(P92558)	66 (84)
	NU2M	membrane domain	–	AtMg00285<br>AtMg01320	-<br>-
	NU3M	membrane domain	–	AtMg00990	-
	NU4M	membrane domain	–	AtMg00580	-
	NU5M	membrane domain	AAW67091	AtMg00060<br>AtMg00513<br>AtMg00665	55 (94)
Complex II	SDH1	Succinate dehydrogenase flavoprotein subunit 1	PITG_18354	At5g66760	69 (99)
	SDH2	Succinate dehydrogenase iron-sulfur protein	PITG_10951	At3g27380	67 (84)
Complex III	Cytochrome c1		PITG_18776	At5g40810	55 (85)
	Rieske protein		PITG_05840	At5g13430	64 (68)
Complex IV	Cox1	Subunit 1	NP_037600	YP_009472108	77 (97)
	Cox2	Subunit 2	NP_037598	YP_009472098	64 (92)
	Cox3	Subunit 3	NP_037612	YP_009472127	60 (85)
Cytochrome c	Cyt c		PITG_12682	At1g22840	68 (89)
Alternative oxidase	AOX 1		PITG_09890<br>PITG_09886<br>PITG_09887	At3g22370	49 (55)<br>46 (56)<br>46 (56)

[1]Selected proteins participating in the *A. thaliana* (Schertl & Braun, 2014) electron transport chain are included. The corresponding *P. infestans* sequences were identified by BLASTp.
[2]A second sequence, PITG_14849, exhibited similarity to the *A. thaliana* alternative NAD(P)H dehydrogenases: 36 (92) to NDA1, 35 (86) to NDA2, and 35 (90) to NDB1. Both PITG_14863 and PITG_14849 are predicted to have mitochondrial location.
[3]These sequences are classified as type II mitochondrial NAD dehydrogenases by (Feng et al., 2012). Note that PITG_14863 exhibits 30 (70) to *Toxoplasma gondii* type II mitochondrial NAD dehydrogenase-1 (XM_002369634).
[4]SwissProt codes for the subunits are given.
[5]We found no homolog for the complex I subunit that binds rotenone (SwissProt code NUCM) (Darrouzet, Issartel, Lunardi, & Dupuis, 1998), although sequences for several of the other complex I core subunits (Berrisford, Baradaran, & Sazanov, 2016) are present.

References

Berrisford, J. M., Baradaran, R., & Sazanov, L. A. (2016). Structure of bacterial respiratory complex I. *Biochimica et Biophysica Acta*, *1857*(7), 892–901. https://doi.org/10.1016/j.bbabio.2016.01.012

Darrouzet, E., Issartel, J. P., Lunardi, J., & Dupuis, A. (1998). The 49-kDa subunit of NADH-ubiquinone oxidoreductase (Complex I) is involved in the binding of piericidin and rotenone, two quinone-related inhibitors. *FEBS Letters*, *431*(1), 34–38. Retrieved from http://www.ncbi.nlm.nih.gov/pubmed/9684860

Feng, Y., Li, W., Li, J., Wang, J., Ge, J., Xu, D., … Yang, M. (2012). Structural insight into the type-II mitochondrial NADH dehydrogenases. *Nature*, *491*(7424), 478–482. https://doi.org/10.1038/nature11541

Schertl, P., & Braun, H.-P. (2014). Respiratory electron transfer pathways in plant mitochondria. *Frontiers in Plant Science*, *5*(April), 1–11. https://doi.org/10.3389/fpls.2014.00163
